# Health inequity drives disease biology to create disparities in prostate cancer outcomes

**DOI:** 10.1172/JCI155031

**Published:** 2022-02-01

**Authors:** William G. Nelson, Otis W. Brawley, William B. Isaacs, Elizabeth A. Platz, Srinivasan Yegnasubramanian, Karen S. Sfanos, Tamara L. Lotan, Angelo M. De Marzo

**Affiliations:** Sidney Kimmel Comprehensive Cancer Center, Johns Hopkins University School of Medicine, Baltimore, Maryland, USA.

## Abstract

Prostate cancer exerts a greater toll on African American men than on White men of European descent (hereafter referred to as European American men): the disparity in incidence and mortality is greater than that of any other common cancer. The disproportionate impact of prostate cancer on Black men has been attributed to the genetics of African ancestry, to diet and lifestyle risk factors, and to unequal access to quality health care. In this Review, all of these influences are considered in the context of the evolving understanding that chronic or recurrent inflammatory processes drive prostatic carcinogenesis. Studies of inherited susceptibility highlight the contributions of genes involved in prostate cell and tissue repair (BRCA1/2, ATM) and regeneration (*HOXB13* and *MYC*). Social determinants of health appear to accentuate these genetic influences by fueling prostate inflammation and associated cell and genome damage. Molecular characterization of the prostate cancers that arise in Black versus White men further implicates this inflammatory microenvironment in disease behavior. Yet, when Black and White men with similar grade and stage of prostate cancer are treated equally, they exhibit equivalent outcomes. The central role of prostate inflammation in prostate cancer development and progression augments the impact of the social determinants of health on disease pathogenesis. And, when coupled with poorer access to high-quality treatment, these inequities result in a disparate burden of prostate cancer on African American men.

## Introduction

In the United States, prostate cancer is one of the most common cancers afflicting aging men (1, 2). Autopsy studies hint that as many as 59% of men aged 80 years and older may harbor carcinomas in their prostates, most of which go unrecognized (3). The American Cancer Society estimates that 1 in 9 men will be diagnosed with prostate cancer in their lifetimes, while 1 in 41 will die from disease progression. Yet despite (or because of) the high incidence and prevalence of the disease, prostate cancer outcomes are plagued with health inequities. Specifically, the disease disproportionately impacts African American men. Over the last decade, Black men suffered a 1.78-fold higher prostate cancer incidence and a 2.2-fold higher prostate cancer mortality than non-Hispanic White men, a disparity larger than for any other common cancer (4). For this reason, this Review focuses on prostate cancer in Black men versus White men and on heritable and environmental factors that modify outcomes.

Differences and disparities in prostate cancer incidence and mortality have long been recognized among men of varied race, ethnicity, socioeconomic status, and place of birth or residence (4). This uneven burden of disease has been attributed to inherited genes influencing disease risk, to diet and lifestyle affecting disease pathogenesis, and to unequal access to high-quality treatment undermining disease outcome (5). Despite knowledge of these factors, the increased prostate cancer incidence and mortality among Black men have not improved, even as prostate cancer mortality overall has declined. Better insights into the excess of life-threatening prostate cancers in Black men that lead to actionable interventions are surely needed.

## Genetics, ancestry, and prostate cancer risk

US government health statistics classify citizens self-declaring as *White* (origins in Europe, the Middle East, or North Africa), *Black or African American* (origins in any of the Black racial groups of Africa), *American Indian or Alaska Native* (origins in peoples of North, South, or Central America who maintain tribal affiliation), *Asian* (origins in the Far East, Southeast Asia, or the Indian subcontinent), or *Native Hawaiian or Other Pacific Islander* (origins in Hawaii, Guam, Samoa, or other Pacific Islands). There is significant admixture in the US population — some 33.8 million people reported being of more than one race in the 2020 US Census. As such, the US government racial categories tend to be more sociocultural than genetic (6–8). To consider the contribution of genetics to disparities in prostate cancer incidence and mortality in the United States between Black and White men, the role of European versus African ancestry must be more directly addressed.

A hereditary component to prostate cancer was first proposed in the 1960s and confirmed by studies of Mormon genealogies (9, 10). Later, statistical analysis of inheritance patterns in families of men with prostate cancer suggested that some cases were attributable to rare high-penetrance genes (11). Nonetheless, despite an intensive search for such genes, and further evidence of a strong genetic influence on prostate cancer detected in twin studies (12), defined genetic risk alleles were difficult to pin down until a reproducible prostate cancer risk was ascribed to chromosome 8q24 (13). Loci at 8q24 inaugurated a list that now includes 269 genetic risk variants for prostate cancer (14).

Through genetic linkage studies of men of European ancestry, variants at *HOXB13*, located at 17q21, were found to specifically affect prostate cancer risk (15). The frequency of a *HOXB13 G84E* variant was higher among affected than unaffected men (1.4% vs. <0.4%) and for men diagnosed at a younger age or with a family history of prostate cancer. *HOXB13* encodes a homeobox transcription factor produced in the spinal cord, hindgut, and urogenital sinus in developing embryos, with persistent expression in adult prostate tissues (16). In prostate cancer cells, HOXB13 interacts with the androgen receptor (AR) to modulate its transcriptional output (17, 18). Though *HOXB13 G84E* was found in studies of men with European ancestry, other founder mutations have been associated with prostate cancer in men of other ancestries: *G132E* for Japanese men (19), and *G135E* for Chinese men (20). The mutations cluster within a conserved domain in the HOXB13 protein responsible for binding to the homeobox cofactor MEIS1, hinting that altered HOXB13-MEIS interactions might contribute to cancer promotion (21). More recently, a stop-loss mutation, *HOXB13 X285K*, was observed in a study of men with prostate cancer who were of African descent in Martinique (22). This African-specific variant, with an additional 95 amino acids in the HOXB13 homeodomain if translated, appears to be associated with prostate cancer at an early age in Black men (23).

There also appear to be inherited contributions to the propensity to develop life-threatening prostate cancer. Aberrations at the DNA repair genes *BRCA1*, *BRCA2*, and *ATM* have been found in as many as 19.3% of metastatic castration-resistant prostate cancers (CRPCs); and among cases with biallelic inactivation of *BRCA2*, approximately half had inherited a mutated inactive copy (24, 25). Mutant DNA repair genes were also more common in Black men than in White men with prostate cancer (26). DNA double-strand break repair gene deficiency in CRPCs, as in breast and ovarian cancers, constitutes an indication for use of poly(ADP-ribose) polymerase (PARP) inhibitors for treatment (27); DNA mismatch repair gene deficiencies serve as an indication for use of immune checkpoint inhibitors (28).

Can African-ancestry genes account for any of the increased burden of prostate cancer among US Black men? Allele –8 of the microsatellite marker DG8S737 on chromosome 8q24 has been associated with a 1.6-fold increased risk of prostate cancer both for men with European and for men with African ancestry (13). However, 30% of Black men carry high-risk 8q24 alleles, while only 13% of White men appear to be carriers, with a population-attributable risk of this allele for Black men estimated at 16% (13). One 8q24 variant (rs72725854) strongly enriched in men of African ancestry has been associated with family history of prostate cancer, early age at diagnosis, and aggressive disease behavior (29). For men with African ancestry (with ~6% allele frequency), this variant may account for 32% or more of familial prostate cancer risk (29). Chromatin conformation capture and dCas9-mediated enhancer blocking studies place the high-risk variant within a prostate cancer–specific enhancer region, serving to modulate expression of *MYC*, *PCAT1*, *PRNCR1*, and other 8q24 genes implicated in prostatic carcinogenesis (30).

The inherited predisposition for prostate cancer implicates prostate cell and tissue repair (DNA double-strand break and mismatch repair genes) and regeneration (*HOXB13* and putative *MYC* enhancer) in disease pathogenesis. Presumably, these functions act in response to carcinogen exposures or chronic inflammatory states (31). Also, African ancestry may underpin some of the disproportionate prostate cancer vulnerability. To exploit knowledge of germline variants and prostate cancer risk, attempts are under way to produce genetic risk score (GRS) tools for use in clinical practice. To be most useful, the GWAS underlying the GRSs must encompass many race and ethnic groups. In a large multi-ancestry meta-analysis of prostate cancer GWAS, the top GRS decile provided odds ratios for disease development of 5.06 for men of European ancestry and 3.74 for men of African ancestry (32). The meta-analysis further revealed that compared with men of European ancestry, men of African ancestry had a mean GRS 2.18 times higher and men of East Asian ancestry 0.73 times lower (32). Current guidelines for prostate cancer screening do not feature GRSs, instead focusing on serum prostate-specific antigen (PSA) testing of men aged 55 to 69 years. Though not recommended by the US Preventive Services Task Force, many Black men are offered screening at a younger age. Perhaps, moving forward, GRSs could help inform who needs screening and at what age, or could be used in conjunction with serum PSA tests to reduce unnecessary biopsies.

## Race/ethnicity, diet and lifestyle, and prostate cancer risk

Diet and lifestyle together exert a dominant influence on prostatic carcinogenesis. Historically, prostate cancer incidence and mortality were higher in the United States and Western Europe than in Asia and Africa (33). Yet migrants from Asia to North America, and descendants of sub-Saharan Africans in the United States and the Caribbean, exhibit incidence and mortality rates as high as, or higher than, those of European American men (33–37). For Asian immigrants to North America, the risk for prostate cancer increased with duration of residence and adoption of dietary habits (38). This does not simply reflect differences in disease detection. Autopsies of men dying of unrelated causes in different parts of the world also show differences in prostate cancer prevalence with age, with less disease in native Africans than in US Black populations (39, 40). These findings suggest that environmental exposures likely affect prostate cancer initiation and progression, or that conditions in lesser developed countries might hinder prostatic carcinogenesis (31). In support of this notion, prostate cancer appears to be on the rise throughout the world accompanying increases in economic prosperity and income inequality, and along with the progressive aging of resident populations (41).

The normal prostate depends on male sex steroid hormones for its development and differentiated function. The major circulating androgenic hormone testosterone is converted in the prostate to the more potent 5α-dihydrotestosterone by 5α-reductase (SRD5A) (42). Both testosterone and 5α-dihydrotestosterone bind to intracellular androgen receptors (ARs) and trigger changes in conformation that allow dissociation from protein chaperones and translocation into the cell nucleus, where AR acts as a ligand-dependent transcriptional regulator for differentiation genes, such as *KLK3* (encoding PSA) (43–45). Because prostate cancers have been recognized for more than 80 years as responding to therapeutic reductions in circulating androgens, a great deal of attention has been afforded to a causative role for androgens in prostate cancer development (46, 47). In this line of thought, increased prostate cancer incidence and mortality in Black versus White men were speculatively attributed to higher levels of circulating sex steroids, to differences in AR structure and function, or to both differences in hormone levels and ancestry-associated *AR* variants (48–51). There are well-known differences in the lengths of CAG and GGN repeats in *AR* among race/ethnic groups associated with differences in transcriptional *trans*-activation and correlated with prostate cancer risk (49, 50, 52).

Subsequent reports have challenged the dogma that prostate cancer is *caused* by excess androgens or androgen action. A large nationally representative study indicated that serum estrogen levels, but not testosterone levels, were higher among Black versus White men in the United States (53). Both Black men and White men given testosterone supplements as they aged had fewer prostate cancers (54). Nonetheless, though androgens may not directly transform normal prostate cells, accumulating data hint that AR signaling may play a role in the progression of transformed prostate cells to invasive carcinoma cells, as indicated by order and timing of molecular events in prostatic carcinogenesis, particularly the propensity for gene fusions leading to AR-regulated ETS family oncogene expression to appear later than *MYC* activation, telomere shortening, and epigenetic gene silencing (55).

Dietary patterns and physical activity likely account for the majority of prostate cancers in developed countries. Fat intake, particularly of animal fat from red meats, has been consistently associated with prostate cancer, while consumption of tomatoes, soy, and other vegetables may protect against the disease (31, 56–60). Food preparation may also play a role: cooking meats at high temperatures leads to the formation of heterocyclic aromatic amine (HAA) carcinogens, both in the meats and in pan drippings (61). Feeding HAAs to rats causes prostate cancer (62). Charbroiling creates polycyclic aromatic hydrocarbon carcinogens that, when ingested, are adducted to prostate cell DNA and increase prostate cancer risk more prominently among Black men (63). Cooking and dietary practices common among Black populations throughout the 20th century in the United States may have increased the risk of prostate cancers and other chronic diseases (64). In the southern United States, the cooking of collard greens features the use of drippings from the pan-frying of pork, ensuring that even vegetable intake includes HAA exposure. In addition, Black individuals often constitute the majority population in low-income urban neighborhoods with limited access to healthy food options (termed “food deserts”), where convenient and affordable food options are often less nutritious and of higher caloric density (65). Low physical activity, particularly in comparison with caloric intake, may create energy imbalance fueling the growth of established prostate cancers (66, 67). Barriers to physical activity for Black individuals in urban neighborhoods with high poverty rates are numerous, ranging from inadequate parks and open spaces to worries about violence (68).

## Race/ethnicity and the molecular pathogenesis of prostate cancers

**Figure 1:**
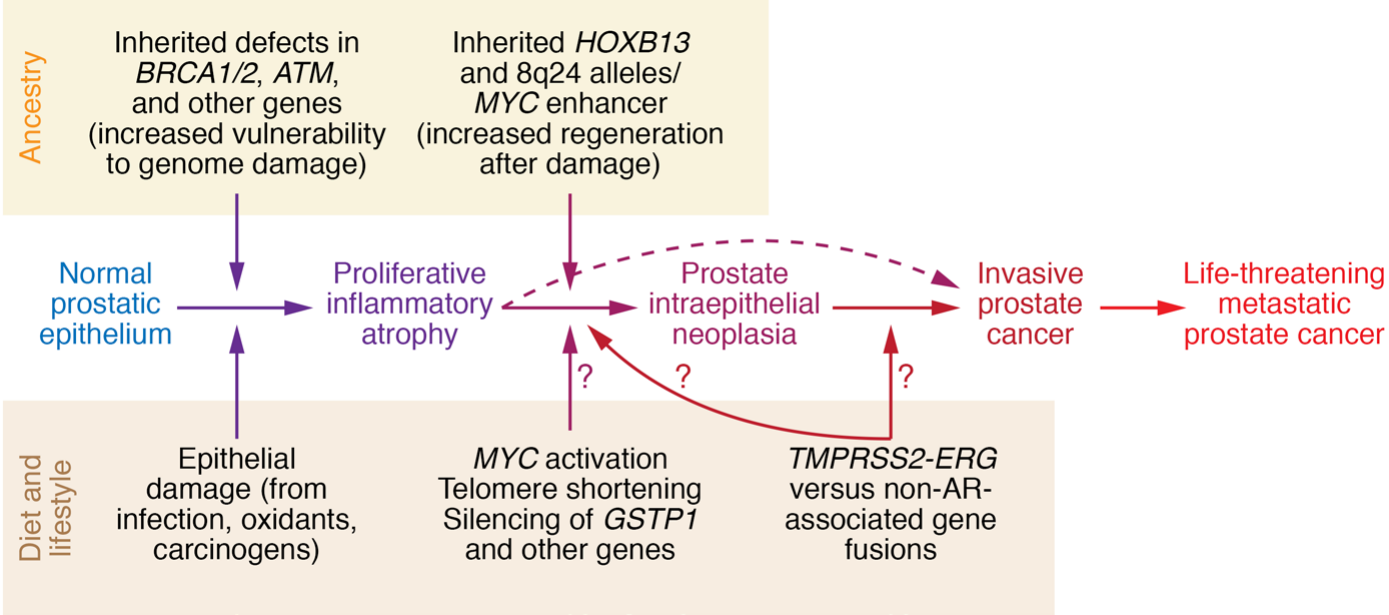
Diet and lifestyle and ancestry converge to produce proliferative inflammatory atrophy to drive the molecular pathogenesis of prostate cancer. Inherited vulnerability to cell and genome damage repair and response sensitizes prostate cells to infections, inflammation, and carcinogens, leading first to proliferative inflammatory atrophy and then to neoplastic transformation and malignant progression. Gene rearrangements could occur via AR-dependent mechanisms, like *TMPRSS2-ERG*, or non-AR-dependent mechanisms.

Prostate cancers carry numerous somatic genome and epigenome alterations that evolve over many years as the disease progresses from initiation to lethal metastatic dissemination (Figure 1 and refs. 69, 70). The earliest changes appear to be (a) activation of MYC, leading to enlarged nuclei and nucleoli; (b) shortened telomere sequences; (c) increased DNA methylation at genes such as *GSTP1* and others with reduced DNA methylation in repeat sequences; and (d) gene rearrangements that activate ETS family transcription factors (56, 71–74). Loss of PTEN and *TP53* mutations more commonly appear in life-threatening metastatic disease (75–78). Genome and epigenome alterations differ from case to case, and between different foci in individual cases (70). In one study, each prostate cancer exhibited a mean of 3866 base mutations (range 3192–5865), 20 non-silent coding sequence mutations (range 13–43), and 108 rearrangements (range 43–213) (79). In another, somatic DNA hypermethylation was found at 5408 regions, with 73% of the sites located near genes (5′, 3′, or intron-exon junctions) and the remaining 27% of the sites at conserved intergenic sequences (80). DNA hypermethylation was maintained through metastatic dissemination up to the time of death (81).

Rearrangements between *TMPRSS2*, encoding an androgen-regulated protease, and *ERG*, an ETS transcription factor, are among the most common somatic alterations in human prostate cancers (70). *TMPRSS2-ERG* and other rearrangements in prostate cancer cells may result from a molecular accident accompanying AR recruitment of the topoisomerase TOP2B to regulatory sequences near target genes during transcriptional activation (73, 82). TOP2B untangling appears to be needed for transcriptional regulatory sequences to adopt a looped conformation in response to AR activation by ligand binding (73). TOP2B acts by catalyzing breakage and rejoining reactions where enzyme subunits transiently link to broken DNA ends; untangling occurs as double-strand DNA molecules pass through the transient breaks. However, under certain conditions, TOP2B-linked breaks can be processed to generate free ends competent for recombination via non-homologous end joining (NHEJ) (83). *TMPRSS2-ERG* rearrangements arise near TOP2B binding sites at each gene (73). Sequencing of *TMPRSS2-ERG* rearrangement junctions revealed sequence microhomologies consistent with NHEJ (84).

Despite the frequent occurrence of rearrangements involving AR-regulated genes in prostate cancers, other sites of DNA breakage and recombination are evident in many cases. An extreme example may be chromothripsis, a chromosome “shattering” phenomenon in which large numbers of rearrangements arise preferentially in cases without ETS gene fusions (85). These rearrangements may not necessarily involve TOP2B, and may instead be driven by inflammatory oxidant stress, bacterial toxin exposure, and/or dietary carcinogens (74, 86). This suggests that there may be at least two dominant influences on prostatic carcinogenesis, one involving androgen signaling and the other promoted by inflammatory genome damage.

Most somatic genome defects are shared between prostate cancers from Black and White men (87). Nonetheless, there have been several reports highlighting potential differences segregating with self-reported race. Such studies have opportunistically exploited biospecimen collections featuring both Black and White cases suitable for genetic, epigenetic, and gene expression analyses, largely comprising prostate biopsy materials, radical prostatectomy specimens, and prostate cancer tissues harvested at autopsy. As a result, the studies present considerable methodologic challenges. Foremost may be biases associated with differences in how prostate cancers are detected and diagnosed among Black versus White men, with Black men less likely to have been diagnosed as a result of screening and more likely to have presented at a higher stage of disease, i.e., to have undergone biopsy or operation later in the natural history of the disease (88). Illustrative of this phenomenon, some somatic genetic changes reported at higher frequency among early-stage prostate cancers in Black men were reminiscent of genetic changes seen in more-advanced-stage prostate cancers in White men (89). Another methodologic impediment is the poor representation of prostate cancer cases in biospecimen collections, despite a willingness of Black men to consent to biospecimen use in prostate cancer biorepositories (90).

With these limitations in mind, several studies have catalogued differences in somatic genotypes, epigenotypes, and phenotypes for prostate cancers from Black versus White men. The most strikingly consistent dissimilarities may be the lower frequencies of *TMPRSS2-ERG* rearrangements, *PTEN* deletions, and *SPOP* mutations in prostate cancers from Black men. An initial study (*n =* 64 Black men) detected *TMPRSS2-ERG* gene fusions in prostate cancers from 50% of White men, compared with 31.3% of Black men and 15.9% of Japanese men (91). In another cohort (*n =* 105 Black men), differences between White and Black men were 42.5% versus 27.6% for *ERG* rearrangements, 19.8% versus 6.9% for *PTEN* deletions, and 10.3% versus 4.5% for *SPOP* mutations (92). In a still larger data set (*n =* 169 Black men), *ERG* and *PTEN* alterations were proportionately less frequent among Black men compared with their White counterparts (25% vs. 51% for *ERG* and 18% vs. 34% for *PTEN*) (93). Provocatively, a small study from South Africa (*n =* 6 Black men) found an absence of *TMPRSS2-ERG* rearrangements and rare *PTEN* losses (94). Low levels of ERG expression were also seen in prostate cancers from men in Ghana and Senegal (95).

A handful of genes other than *SPOP* may be mutated more frequently in prostate cancers from Black men. Whole exome sequencing of localized prostate cancers from Black men (*n =* 102) found that 5% of the cases carried loss-of-function mutations in *ERF*, an ETS transcriptional repressor (96, 97). Further analysis in cell culture revealed that ERF knockdown produced a gene expression signature reminiscent of oncogenic *ERG* activation (96). In a large study of acquired genetic defects (*n =* 171 Black men), of the top 22 genes mutated in prostate cancers from Black men, only two were found more commonly than in cancers from White men, *ZMYM3* (11.7% vs. 2.7%) and *FOXA1* (11.7% vs. 5.4%), while mutations at genes like *SPOP* and *TP53* were less often present (98). Mutations affecting *ZMYM3*, encoding a regulator of BRCA1 function, seemed to correlate with widespread unbalanced allele frequencies in the cancers from Black men, suggesting that losses of *MAP3K7*, *RB1*, *BNIP3L*, *THADA*, and *NEIL3* and gains of the genomic region encompassing *MYC* might underlie aggressive disease behavior (98, 99).

Hypermethylation of a CpG island at the *GSTP1* promoter leading to loss of GSTP1 expression in prostate cancer was one of the first examples of epigenetic gene silencing in human cancers (100). Since its first report, *GSTP1* silencing has remained the earliest and most frequent gene function defect in prostate cancer, affecting more than 90% of cases (101). Now, *GSTP1* hypermethylation assays are approved and marketed as adjuncts to prostate cancer diagnosis, both for Black and for White men (102). Absence of GSTP1 sensitizes prostate cancer cells to mutagenic damage by HAA carcinogens, and confers improved survival, despite increased genome damage, in response to chronic oxidant stress (103, 104). Recently, attention has focused on prostate cancer cases in which GSTP1 expression is retained (105). In prostatectomy tissues from Black and White men, GSTP1^+^ prostate cancer was overrepresented among tumors from Black men (9.5% vs. 3.2%) (105). As has been seen with somatic genetic alterations, different changes in CpG dinucleotide methylation across the epigenome have been reported for more aggressive versus more indolent prostate cancer behavior in Black men (*n =* 76) (106). Provocatively, an analysis of 190 metabolites across prostate cancer versus non-cancerous prostate tissues from African ancestry–verified Black men (*n* = 33) identified increases in methionine and homocysteine that could affect biologic methylation reactions, a phenomenon evident in the plasma and more prominent than for White men with prostate cancer (107). Whether the observed increases can explain the reported differences in DNA methylation has not been ascertained.

In comparative gene expression studies, a reproducible finding has been increased expression of immune-related genes in tumors from Black compared with White men. Chronic or recurrent prostate inflammation is likely an important driver of neoplastic transformation and malignant progression in the gland (31, 108). The repeated finding of gene expression differences involving immune response genes hints that there may be an even greater contribution of inflammatory processes to prostatic tumorigenesis in Black men than in White men (109–115). This hypothesis is supported by studies demonstrating a distinct immune tumor microenvironment present in prostate cancers arising in Black men, characterized by an increase in plasma cells, evidence of NK cell activity, and higher IgG expression (116). However, more work is needed to clarify mechanisms by which these inflammatory processes promote or protect against prostate cancer pathogenesis and progression to life-threatening metastatic disease.

Another pathway in prostate cancers from Black men illuminated by gene expression analysis is lipid metabolism (111). Both fatty acid synthase (FASN) and its upstream regulator MNX1 appear to be preferentially upregulated in tumors from Black men versus White men (111, 117). This clearly provides tantalizing associations with dietary influences on prostate cancer initiation and progression, though the mechanisms by which augmented lipid metabolism promotes malignant phenotypes remain to be elucidated.

In all, the molecular characterization of prostate cancer genes and gene function hints that several distinct molecular subsets (ERG^+^ vs. ERG^–^, PTEN^+^ vs. PTEN^–^, GSTP1^+^ vs. GSTP1^–^) may be more or less prevalent among prostate cancer cases in Black versus White men. Immunologic differences associated with self-reported race are also repeatedly observed in localized prostate cancers. Perhaps highly inflamed prostate tissues spawn prostate cancers less likely to contain rearrangements involving AR target genes, a hypothesis and correlation that should be tested. Nonetheless, whether the reported findings reflect differences in disease pathogenesis attributable to inherited African-ancestry genes or to differences in lifestyle and exposures has not been ascertained.

## Inflammation as a driver of prostatic carcinogenesis

Prostate inflammation is as ubiquitous among aging men in the United States as prostate cancer. Inflammatory processes affect both the transition zone of the gland, where symptomatic benign prostatic hyperplasia arises, and the peripheral zone, where prostate cancers appear (108). Yet since peripheral zone prostatitis and early prostate cancer tend to be asymptomatic, epidemiology studies of the two conditions have proven difficult. Since inflammatory damage to the prostate epithelium and prostate cancer can both raise serum PSA levels, when prostate biopsies conditioned on serum PSA elevations are used to test correlations between prostatitis and prostate cancer, the inferred associations are prone to “collider stratification” bias (118). This type of bias can either falsely hint at an association or even incorrectly suggest an inverse association (118). To minimize this methodologic hindrance, study cohorts in which men underwent prostate biopsy without a clinical indication per se have been examined, such as in the placebo arms of the Prostate Cancer Prevention Trial (PCPT) and ensuing Selenium and Vitamin E Cancer Prevention Trial (SELECT) (119, 120). In PCPT, prostate inflammation was correlated with prostate cancer, at an odds ratio of 1.78 for total prostate cancer and 2.24 for high-grade disease (121). For men in the placebo arm of PCPT without prostate cancer on the end-of-study biopsy who enrolled in SELECT, the odds of developing prostate cancer on a second biopsy a mean of 5.9 years later were increased 1.6-fold, depending on the amount of inflammation seen on the first biopsy (122).

**Figure 2:**
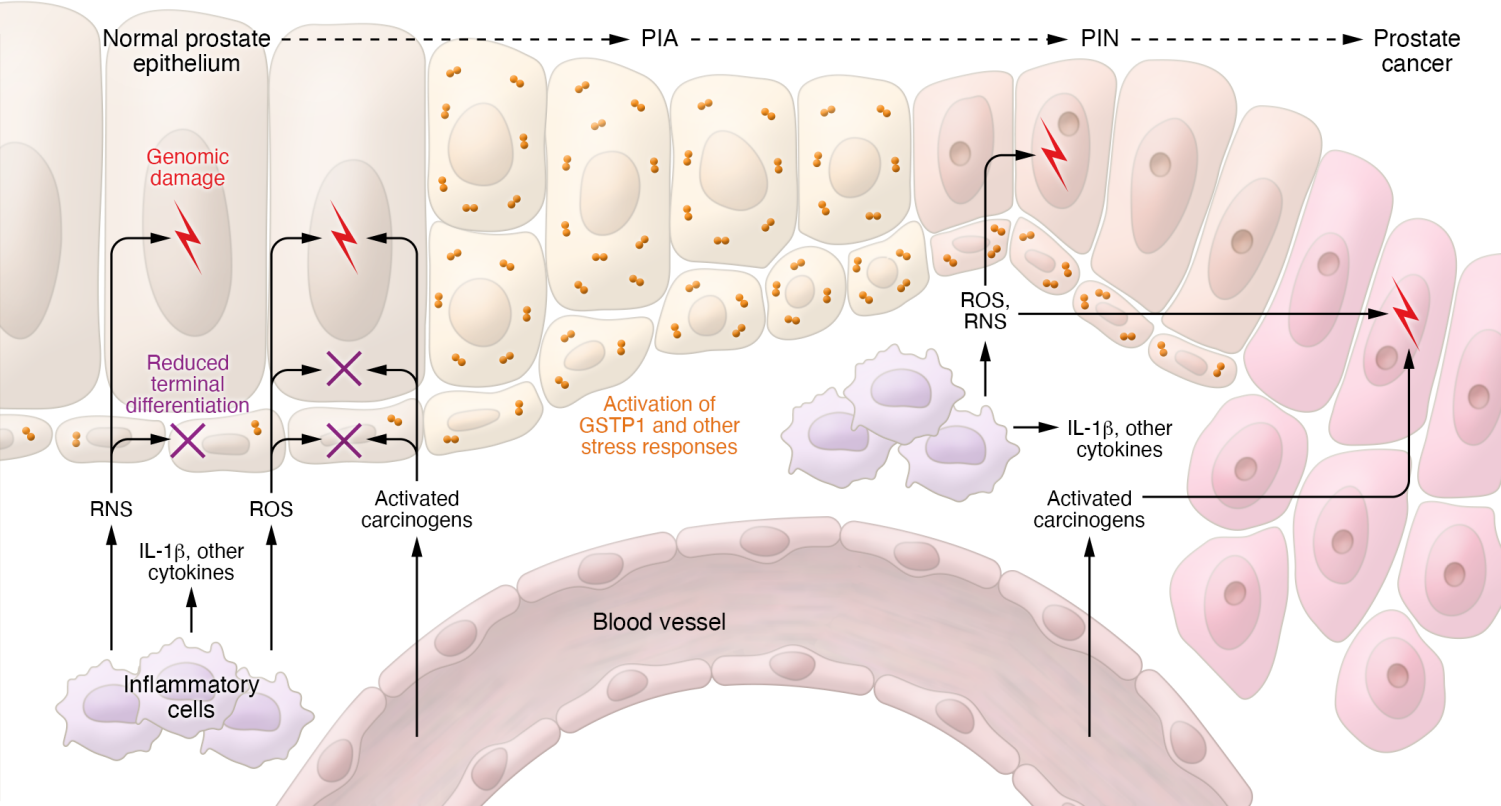
Pro-carcinogenic prostate microenvironment. Prostate epithelium is assaulted by inflammatory ROS and reactive nitrogen species (RNS), by activated dietary carcinogens, and by inflammatory cytokines. The result is cell and genome damage leading to activation of stress response pathways, reduced terminal differentiation, and regenerative proliferation characteristic of the prostate cancer precursor proliferative inflammatory atrophy. Adapted with permission from the *New England Journal of Medicine* (31).

Inflammation likely promotes prostatic carcinogenesis via the generation of proliferative inflammatory atrophy (PIA) lesions, distinct precursors to prostatic intraepithelial neoplasia (PIN) and prostate cancer (Figure 2 and refs. 123–125). PIA cells typically exhibit arrested differentiation, activation of stress response pathways, and regenerative proliferation, and often share genetic and epigenetic alterations with prostate cancer (126–134). The most compelling evidence for PIA involvement in prostatic carcinogenesis may be the consistent finding of PIA lesions arising before prostate cancer in rodent models of the disease induced by exposures, including estrogens and dietary carcinogens (108). Production of inflammatory cytokines, like IL-1β, IL-6, and IL-8, frequently appears in and around PIA lesions (135, 136). Mice engineered to produce IL-1β in the prostate exhibited acute and chronic inflammation, epithelial changes reminiscent of human PIA, and production of downstream proinflammatory cytokines (137). One such cytokine may be macrophage inhibitory cytokine-1 (MIC-1), reported to be increased in the serum and urine of African American men with prostate cancer (138).

Though the inflamed microenvironment that begets PIA, PIN, and prostate cancer has not been fully characterized, both innate and adaptive immune infiltrates are evident. CD4^+^ T cells recovered from the prostate tissues skew toward a Treg or a Th17 phenotype, while oligoclonal CD8^+^ T cells express PD-1 (139, 140). Some infiltrating T cells recognize peptides derived from prostate proteins, including PSA and prostate stem cell antigen (PSCA), though the full spectrum of antigens driving T cell responses has not been determined (141–143). The contributions of these T cells to the maintenance of chronic prostate inflammation, or to the “immunoediting” of neoplastic cells spawned by PIA lesions, have not been elucidated. In one mouse prostate cancer model, supplementation of CD4^+^ Tregs reduced inflammatory cytokine production in the prostate and led to fewer prostate cancers, while CD4^+^ Treg depletion had the opposite effect (144). Inflamed prostate tissues contain abundant immune cells, including neutrophils, macrophages, and mast cells, that express 1L-17 (145, 146). Neutrophils appear to be the source of the numerous corpora amylacea in inflamed prostate tissues readily familiar to prostate pathologists: a proteomics analysis revealed the microscopic bodies to be composed of calprotectin, myeloperoxidase, and α-defensins, all characteristically present in neutrophil granules (147). Corpora amylacea have been described along with PIA-like lesions in aging rats prone to chronic inflammation and prostate cancer (148). In one study of more than 1300 cases cataloguing immune cell infiltrates in prostate cancers, increased numbers of plasma cells, IgG expression, and NK cell activity were found in the tumor microenvironment in Black versus White men, findings that portended improved recurrence-free survival after prostate surgery for localized disease (116).

Prostate infections have long been proposed as stimuli for prostatic inflammation and, in turn, as risk factors for prostate cancer. Several microorganisms (bacterial, viral, and parasitic) have been investigated in relation to prostate cancer risk (149–151). A notable limitation to linking microorganisms in the prostate at the time of cancer diagnosis to the risk of prostate cancer development is that the initiating infection may have occurred many years previously (152). Likewise, contrary to the dogma of Koch’s postulates requiring a pathogen to be present with the disease, the collateral damage caused by a prostate infection may be what promotes cancer, with the offending pathogen cleared many years before cancer became evident. With this in mind, prostate infection and inflammation raise PSA levels in young men, and Black men aged 20 to 45 years have been reported to have higher PSA levels than White men (149, 153). PSA values in midlife are a harbinger of future life-threatening prostate cancer (154).

## Ancestry, inequity, and biology modify prostate cancer outcomes

Confronting disparities in prostate cancer outcomes for Black men versus other race/ethnic groups in the United States, prostate cancer researchers have long grappled with whether they reflect genetic predisposition from African ancestry, environmental conditions disproportionately affecting Black men, or lower quality and accessibility of health care available to various minority groups and low-income populations generally. The findings presented in this Review hint that the impact of inheritance, the environment, and sociology on the etiology of prostate cancer and its propensity to threaten life has progressively converged on a unifying mechanism. Diet and lifestyle promote prostate inflammation, generating PIA lesions poised to birth prostatic adenocarcinomas (108). Genetic predisposition, particularly variants in genes for genome damage and repair and/or cell and tissue damage and regeneration, augments the consequences of the molecular mayhem inflicted on the prostate by ROS and carcinogens. The resultant gene-environment interactions conspire to produce more prostate cancers and more disease virulence. These mechanisms are amplified by inequities associated with poverty and a history of racism.

The Centers for Disease Control and Prevention defines social determinants of health as “conditions in the places where people live, learn, work, and play” (155). With the dominant influence of diet and lifestyle on the molecular pathogenesis of prostate cancer, the social determinants of health collude to promote disparities in prostate cancer between Black and White men and to undermine health care access, quality, and equity for Black men. An accumulating body of evidence suggests that health care quality has a significant impact on outcomes of all cancers in vulnerable populations in the United States (156). Prostate cancer is no exception: when prostate cancer care is received at a Veterans Health Administration facility or as part of a clinical trial, Black men and White men exhibit similar outcomes when receiving the same treatment for the same stage of disease (157–161). And, in a study of prostate cancer–specific survival among Black and White men in Detroit, though Black men exhibited worse outcomes, adjustment for socioeconomic status eliminated the survival differences (162).

## Implications

**Figure 3:**
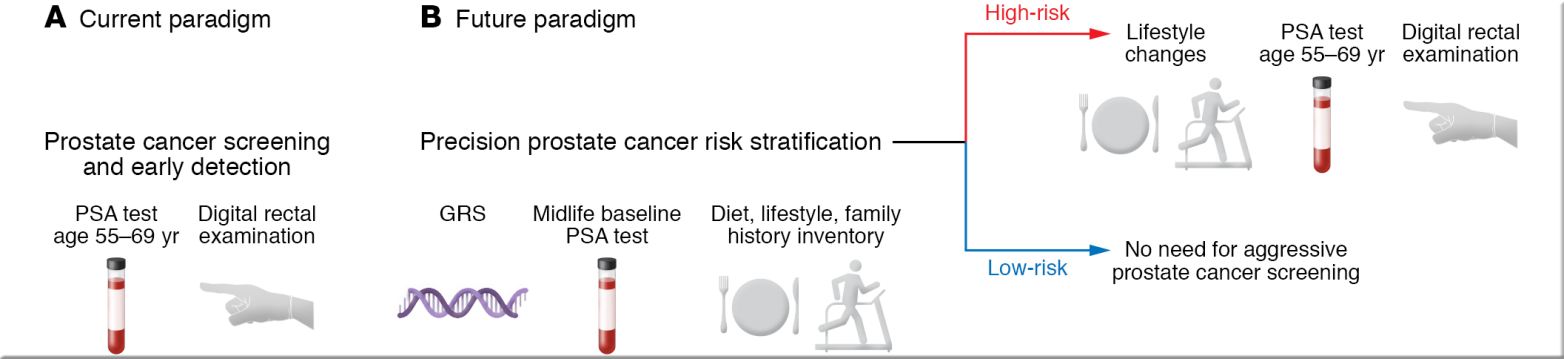
Movement toward a proactive prostate cancer risk stratification approach to disease control for improvement of prostate cancer mortality and elimination of disparities. (**A**) Guidelines differ as to when men in the general US population should undergo prostate cancer screening via PSA test and digital rectal examination; and how Black men should best be screened has also been debated (165–167). Screening detects prostate cancer at an early stage, at a cost of significant overdiagnosis and overtreatment. The use of active surveillance approaches for low-risk prostate cancer to mitigate this problem is not evenly distributed between White and Black men or among men of higher versus lower socioeconomic status (167). (**B**) A future paradigm might feature risk stratification, using germline genetic testing (for risk alleles associated with European or African ancestry), midlife PSA testing, and an inventory of diet, lifestyle, and family history. Men at high risk could be steered toward more vigilant prostate cancer screening regimens and coached to pursue substantive dietary modification, weight loss, and exercise, while men at low risk might not need such aggressive intervention. Prostate cancer screening itself is somewhat limited by health care access, and this new precision paradigm of risk ascertainment and intervention could be even more sensitive to social determinants of health and health care inequities.

To overcome the excess burden of prostate cancer mortality among Black versus White men in the United States, the social determinants of health and health equity must be more deliberately and directly targeted — a strategy likely to reduce prostate cancer death rates for men of all races/ethnicities. Prostate cancer risk reduction should involve promoting and delivering healthier diets throughout life, particularly among low-income people in both rural and urban areas. Prostate cancer screening, detection, diagnosis, and treatment will likely all benefit from the introduction of a growing body of precision medicine tools, including germline gene testing and better measures of environmental exposures, able to stratify the right man for the right intervention at the right time (Figure 3). Risk-stratified prostate cancer screening must be made available to all men in the United States. The onus of prostate cancer care providers is to ensure that these precision medicine tools are available equitably, such that they ameliorate, rather than exacerbate, the existing disparities borne by Black men (163, 164).

## Version 1 02/01/2022

Electronic publication
